# Large Language Models in Perioperative Antithrombotic Management: A Blinded Scenario-Based Expert Evaluation

**DOI:** 10.3390/diagnostics16172848

**Published:** 2026-09-04

**Authors:** İrem Durmuş, Merve Bulun Yediyıldız

**Affiliations:** Department of Anesthesiology and Reanimation, Kartal Dr. Lütfi Kırdar City Hospital, University of Health Sciences Turkey, 34865 Istanbul, Turkey; mervebulun@gmail.com

**Keywords:** artificial intelligence, decision support systems, perioperative care, anesthesia, anticoagulants, risk assessment

## Abstract

**Background**: Perioperative antithrombotic management is a complex and high-risk aspect of anesthesia practice, requiring a careful balance between bleeding and thromboembolic risks. Large language models (LLMs) are increasingly explored for clinical decision support, but their reliability in complex perioperative antithrombotic scenarios remains uncertain. **Methods**: In this blinded, scenario-based study, 100 elective surgical cases, including grey-zone scenarios, were developed to reflect clinically relevant perioperative antithrombotic decisions. The European Society of Anaesthesiology and Intensive Care/European Society of Regional Anesthesia and Pain Therapy (ESAIC/ESRA) 2022 guideline was incorporated into the standardized prompt as a common framework for neuraxial safety and relevant antithrombotic interruption intervals. Four LLMs (GPT-5.4 Thinking, Claude Opus 4.5, DeepSeek v3.2 Reasoning and Qwen3.5-Plus) were evaluated using a standardized prompt. Model responses were presented without model identity and independently assessed by two experienced anesthesiologists, neither of whom was an author of the present study, across five clinical domains on a 5-point Likert scale, with a separate clinical applicability assessment. **Results**: Using a uniform four-domain composite across all 100 scenarios, overall expert-rated performance differed across models (Friedman χ^2^ = 28.75, *p* < 0.001; Kendall’s W = 0.096). Claude had the highest median primary composite score, although its difference from DeepSeek was not statistically significant. Leave-one-domain-out sensitivity analyses showed that between-model separation was particularly sensitive to the clinical-rationale domain; exclusion of this domain reduced Kendall’s W to 0.030. A secondary five-domain analysis restricted to the 51 common-proceed scenarios, all of which were standard rather than grey-zone cases, also showed an overall between-model difference (Friedman χ^2^ = 55.13, *p* < 0.001; Kendall’s W = 0.360). Because each scenario was queried only once, fine-grained differences in model ordering should be considered provisional. Although all models performed well in drug discontinuation decisions, greater variability was observed in discontinuation timing, anesthesia choice and clinical reasoning. Postponement behaviour varied substantially across models, with DeepSeek and Qwen recommending postponement more often than ChatGPT and Claude. **Conclusions**: LLMs show promise as supportive tools in perioperative decision-making, but their performance remains variable, especially in complex situations. At present, they should be used cautiously and always under expert supervision, rather than as independent decision-makers.

## 1. Introduction

Perioperative management of antithrombotic therapy represents one of the most complex areas of clinical decision-making in modern anesthesia and surgical practice. With increasing life expectancy and the growing prevalence of cardiovascular diseases, the number of patients receiving anticoagulant and antiplatelet therapy has risen substantially [1]. When planning surgery in this patient population, clinicians must carefully balance the need to minimize bleeding risk while simultaneously preventing thromboembolic events [2]. This delicate balance often requires clinical judgement beyond standard algorithms, as decisions are influenced by both patient-specific factors and surgical characteristics.

This complexity becomes even more pronounced in the context of neuraxial anesthesia. Although rare, spinal or epidural hematoma in patients receiving antithrombotic therapy can lead to devastating complications, including permanent neurological injury [3]. For this reason, detailed guidelines have been developed regarding drug discontinuation intervals, timing of resumption and the safety of neuraxial procedures.

However, in real-world clinical practice, “grey-zone” scenarios where guideline recommendations cannot be directly applied are frequently encountered, particularly in patients with multiple comorbidities or overlapping risk factors. In such situations, decisions are shaped not only by guidelines but also by clinician experience and individualized risk assessment [4]. This variability can lead to differences in clinical decisions even among experts and may limit standardization.

In recent years, artificial intelligence and, in particular, large language models (LLMs), have emerged as promising tools in clinical decision support. These models, trained on extensive medical data, are capable of generating contextually relevant responses and, in some cases, demonstrating human-like reasoning processes [5]. Indeed, previous studies have shown that LLMs can achieve performance levels comparable to physicians in certain knowledge-based assessments [6]. Nevertheless, their reliability in high-risk, guideline-driven clinical scenarios remains insufficiently explored. Perioperative antithrombotic management represents a critical test case in this regard, as it requires precise risk balancing and carries significant implications for neuraxial safety. While the general medical knowledge and reasoning capabilities of LLMs have been studied, evidence specifically addressing their performance in anesthesia practice particularly in the context of neuraxial safety is still limited.

This study compared the expert-rated clinical decision-making performance of LLMs in perioperative antithrombotic management using standardized elective surgical scenarios. ESAIC/ESRA 2022 provided a common reference for neuraxial safety, while experienced anesthesiologists evaluated drug discontinuation, timing, surgical management, anesthesia technique, and clinical justification. In addition, variations in model performance were analyzed in grey-zone scenarios where guideline recommendations are less directly applicable.

## 2. Materials and Methods

### 2.1. Study Design

This study was designed as a scenario-based, cross-sectional, blinded and comparative evaluation. The primary aim was to compare expert-rated clinical appropriateness and decision quality across LLM outputs and assess inter-rater consistency.

The study was conducted using a simulation-based approach built on hypothetical clinical scenarios without the use of real patient data. Accordingly, the findings are intended to evaluate the potential role of these models in clinical decision support and educational settings, rather than their direct application in clinical practice.

### 2.2. Case Set Development

A total of 100 elective surgical scenarios were developed for this study. No formal a priori power calculation was performed. The 100-scenario target was chosen pragmatically before model querying to cover antithrombotic classes, treatment indications, surgical contexts, renal and laboratory profiles, and clinically complex cases while keeping blinded expert evaluation feasible. All scenarios were systematically designed to represent clinically relevant situations in perioperative antithrombotic management.

During the scenario development process, multiple variables were considered, including the type of antithrombotic agent used (e.g., warfarin, direct oral anticoagulants and aspirin), the indication for therapy (e.g., atrial fibrillation, deep vein thrombosis and pulmonary embolism), duration of use, the type and bleeding risk of the planned surgery, laboratory parameters (International normalized ratio (INR), platelet count and glomerular filtration rate) and associated comorbidities.

Initial scenario drafts were generated with Gemini 3 Pro (Google, Mountain View, CA, USA) and reviewed and refined before model querying by an experienced anesthesiologist who was not an author of the present study and did not participate in response evaluation. Review focused on clinical realism, internal consistency, and alignment with the intended guideline-based perioperative antithrombotic framework. The final case set therefore comprised expert-refined rather than unedited AI-generated vignettes.

Grey-zone status was assigned during initial case-set generation, before model querying or expert rating, and was therefore independent of subsequent outputs and scores. It identified scenarios in which multiple clinical factors made a single straightforward recommendation less clear and required integration of bleeding and thromboembolic risk, laboratory results, renal function, and surgical context. No formal checklist or scoring algorithm was used. The final set included 48 grey-zone and 52 standard scenarios.

As no real patient data or identifiable information were used, ethical approval was not required for this study.

### 2.3. Model Evaluation Procedure

Each of the 100 clinical scenarios was independently presented to four large language models: GPT-5.4 Thinking (ChatGPT; OpenAI, San Francisco, CA, USA), Claude Opus 4.5 (Anthropic, San Francisco, CA, USA), DeepSeek-V3.2 in reasoning mode (DeepSeek, Hangzhou, China), and Qwen3.5-Plus (Alibaba Group, Hangzhou, China). Model querying was conducted between February and March 2026. All interactions were performed through the respective consumer web interfaces rather than APIs, using the default interface settings. No external web search or tool use was intentionally invoked during model evaluation. Account-level memory and personalization settings were not independently verified at the time of data collection. The model names and modes reported here correspond to the provider-published model nomenclature and/or the options displayed in the respective consumer interfaces at the time of data collection. Because API access was not used, provider-specific backend build identifiers, API snapshot identifiers, temperature values, and random seeds were not available and therefore could not be recorded or controlled.

To ensure methodological consistency and minimize contextual bias, each scenario was submitted in a newly initiated, separate chat session using a standardized prompt. Each scenario was queried once per model, and the first complete response was retained; no regeneration, response selection, or additional prompting was used. No additional explanations or guidance were provided, and the same standardized prompting procedure was applied to all models. The prompt was structured to reflect a preoperative assessment and included patient demographics, comorbidities, indication for antithrombotic therapy, laboratory parameters and details of the planned surgical procedure. The full prompt used in the study is provided as Supplementary Materials.

The models were instructed to generate responses in accordance with the European Society of Anaesthesiology and Intensive Care/European Society of Regional Anesthesia and Pain Therapy (ESAIC/ESRA) 2022 guidelines and from the perspective of an experienced anesthesiologist [7]. Accordingly, each model was expected to provide a structured response including decisions on whether to discontinue antithrombotic therapy, the recommended discontinuation interval if applicable, whether to proceed with or postpone surgery, the preferred anesthesia technique (general or neuraxial) and the clinical justification supporting these decisions.

The ESAIC/ESRA reference primarily standardized neuraxial safety and relevant antithrombotic interruption intervals. Broader perioperative decisions, including treatment interruption and whether to proceed with surgery, were left to each model’s integration of bleeding and thromboembolic risk, laboratory findings, comorbidities, and surgical factors.

Model outputs were retained in their original form; explicit model identifiers were removed before responses were presented to evaluators.

### 2.4. Scoring Framework

Model outputs were scored using a 5-point Likert scale (1–5) specifically developed to assess clinical decision quality. This scale was structured to reflect the clinical appropriateness and safety of the recommendations, where 1 indicated completely inappropriate, 2 inappropriate, 3 borderline or acceptable, 4 appropriate, and 5 fully appropriate.

The evaluation process covered five key decision domains representing the core components of perioperative antithrombotic management. These included the decision to discontinue antithrombotic therapy, the recommended discontinuation timing, the decision to proceed with or postpone surgery, the choice of anesthesia technique, and the quality of the clinical justification provided.

In addition, to assess the real-world applicability of each model’s recommendation, evaluators were asked a binary question: “Would you apply this recommendation in clinical practice?” Responses were recorded as yes or no. This approach allowed for a more comprehensive assessment of model performance, capturing not only theoretical accuracy but also practical clinical applicability.

### 2.5. Human Rating

Model responses were evaluated by two independent anesthesiologists, each with at least 10 years of clinical experience. Neither evaluator was an author of the present study. Assessors were unaware of model identity and had no access to each other’s ratings. For each clinical scenario, the four anonymized model responses were presented in independently randomized order to each evaluator before evaluation proceeded to the next scenario.

Because anesthesia type was scored only when surgery was recommended to proceed, domain counts differed. Each evaluator completed 1903 ratings: 1600 across the four universally applicable domains and 303 anesthesia-type ratings (ChatGPT *n* = 97, Claude *n* = 100, DeepSeek *n* = 53, Qwen *n* = 53), for 3806 ratings overall. The five domains assessed clinical appropriateness rather than formal guideline concordance. Neuraxial safety decisions were judged against established antithrombotic principles; broader perioperative decisions were judged for overall clinical appropriateness and safety based on expert anesthesiology practice.

Before formal evaluation, both evaluators reviewed five sample cases excluded from the study to familiarize themselves with the scoring framework and anchors. This was a qualitative exercise; no inter-rater statistic was calculated, the pilot ratings were not analyzed, and no documented changes were made to the five-domain structure or 1–5 anchors.

### 2.6. Statistical Analysis

All statistical analyses were performed using IBM SPSS Statistics version 29.0 (IBM Corp., Armonk, NY, USA). Two-sided *p* values < 0.05 were considered statistically significant unless otherwise specified.

Inter-rater agreement was assessed separately by model and domain. Exact agreement and unweighted Cohen’s kappa were calculated for all domains. For the ordinal domains, quadratic-weighted kappa was additionally reported to account for the magnitude of disagreement. Absolute agreement was assessed with ICC(2,1) using a two-way random-effects, absolute-agreement, single-measures model, with 95% CIs from 10,000 case-level bootstrap resamples. Anesthesia-type analyses included only cases scored by both evaluators.

Given the ordinal distributions, domains were summarized by median, IQR, and full score distribution. Because the rating distributions were strongly concentrated at the scale extremes, the proportion of ratings judged appropriate (scores 4–5) was also reported for each model and domain, with 95% confidence intervals obtained from 10,000 case-level bootstrap resamples that preserved the paired evaluator ratings within each scenario. An additional descriptive analysis quantified midpoint-straddling disagreements, defined as one evaluator assigning a score of 1–2 and the other assigning a score of 4–5. Because no prespecified safety-error taxonomy was available, a post hoc exploratory descriptive review was performed for responses associated with score-1 ratings. This analysis was not intended to classify hallucinations, guideline deviations, critical errors, or potential harm. Instead, it was used to identify recurring observable response patterns and to select representative examples for supplementary presentation. Medication-discontinuation differences were tested with Cochran’s Q followed by pairwise exact McNemar tests with Bonferroni correction. For discontinuation timing, the decision to proceed with or postpone surgery, and clinical rationale, the two evaluator ratings were first averaged within each scenario to obtain one paired domain score per model. Models were then compared using the Friedman test, followed by pairwise Wilcoxon signed-rank tests with Bonferroni adjustment for the six model-to-model comparisons.

For the primary between-model analysis, a uniform scenario-level composite score was calculated from the four domains applicable to all scenarios: medication discontinuation, discontinuation timing, the decision to proceed with or postpone surgery, and clinical rationale. For each scenario, the two evaluator ratings were first averaged within each domain, and the resulting four domain scores were then averaged to obtain one composite score per model. Anesthesia type was excluded from the primary composite because it was applicable only when a model recommended proceeding with surgery. As a secondary analysis, a five-domain composite including anesthesia type was calculated for the 51 scenarios in which all four models recommended proceeding with surgery. To assess whether the primary composite was disproportionately influenced by any single domain, a leave-one-domain-out sensitivity analysis was performed by recalculating the composite after sequentially excluding each of its four constituent domains. Each resulting three-domain composite was compared across models using the same Friedman test followed by Bonferroni-adjusted pairwise Wilcoxon signed-rank tests. Models were compared using the Friedman test, followed by pairwise Wilcoxon signed-rank tests with Bonferroni adjustment. Bonferroni-adjusted *p* < 0.05 was considered statistically significant. Wilcoxon effect sizes were calculated using Rosenthal’s convention, r = |Z|/√N_total, where N_total represented the total number of observations contributing to each paired comparison (i.e., twice the number of paired scenarios: N_total = 200 for the primary 100-scenario analysis and N_total = 102 for the 51-scenario secondary analysis).

Clinical applicability was recorded independently by each evaluator as yes/no. Inter-rater agreement for this binary item was assessed using exact agreement and unweighted Cohen’s kappa. Applicability was compared across models with Cochran’s Q; significant tests were followed by pairwise exact McNemar tests with Bonferroni adjustment. Ratings were not collapsed into consensus because no adjudication rule was predefined; pooled proportions are descriptive only. Grey-zone versus standard overall scores were explored separately for each model with Mann–Whitney U tests and effect sizes; the four model-specific *p* values were Bonferroni-adjusted, with adjusted *p* < 0.05 considered significant.

The primary four-domain composite analysis was considered the main confirmatory between-model comparison. All domain-specific comparisons, the secondary five-domain composite, clinical-applicability analyses, grey-zone comparisons, leave-one-domain-out sensitivity analyses, and score-1 characterizations were considered secondary or exploratory analyses. Multiplicity adjustments were applied separately within each family of pairwise comparisons rather than across all analyses in the study.

Due to variability in surgical decision-making across models, anesthesia type evaluations were conducted using a two-step approach. First, analyses were performed within each model’s own set of cases where surgery was continued. Second, to ensure comparability across models, a subgroup analysis was conducted including only cases in which all models recommended proceeding with surgery.

Reporting was reviewed against the TRIPOD-LLM guideline [8] and the completed checklist is provided as a Supplementary File S11.

## 3. Results

### 3.1. Inter-Rater Agreement

Inter-rater agreement was generally strong across models and domains. Medication discontinuation showed perfect agreement across all models. Across the full set of domains, unweighted kappa ranged from 0.393 to 1.000, while quadratic-weighted kappa for the ordinal domains ranged from 0.627 to 0.994. The lowest unweighted and weighted kappa values were observed for DeepSeek anesthesia type (uκ = 0.393; wκ = 0.627), for which ICC(2,1) was 0.631 (95% CI 0.429–0.780) (Table 1). Across 1903 evaluable scenario-domain pairs, 12 (0.6%) showed midpoint-straddling disagreement between evaluators, and only one pair (0.05%) represented an extreme 1-versus-5 disagreement.

For the binary clinical-applicability assessment, exact agreement was 95.0% for ChatGPT, 88.0% for Claude, 85.0% for DeepSeek, and 93.0% for Qwen, with corresponding unweighted kappa values of 0.857, 0.759, 0.704, and 0.861, respectively (Table 1).

### 3.2. Descriptive Statistics and Performance Distribution

**Figure 1:**
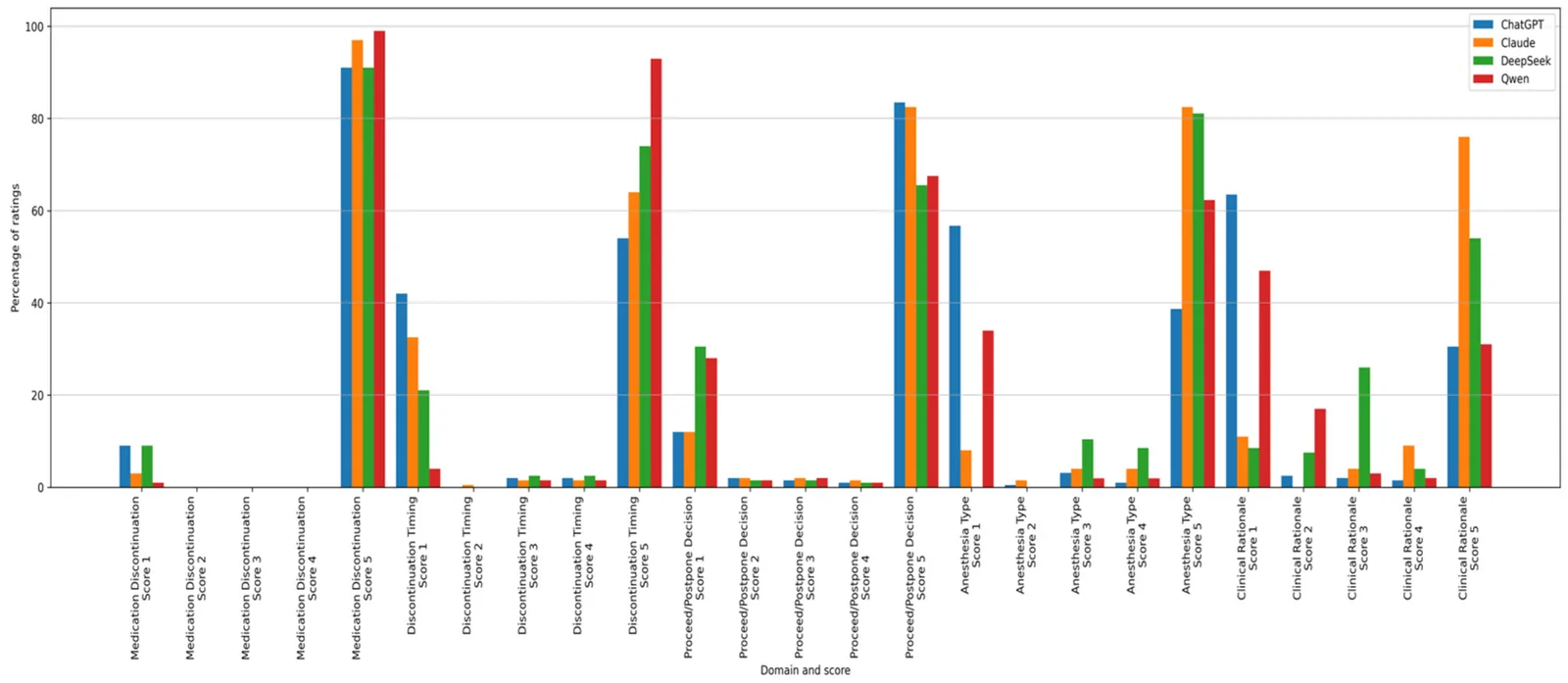
Distribution of expert ratings across clinical decision domains by model. Grouped bars show the percentage distribution of individual evaluator ratings across scores 1–5. Score 1 indicates completely inappropriate and score 5 fully appropriate. All domains included 200 ratings per model (100 scenarios × 2 evaluators), except anesthesia type, which included ChatGPT *n* = 194, Claude *n* = 200, DeepSeek *n* = 106, and Qwen *n* = 106 ratings. Percentages are based on paired evaluator-level ratings and therefore do not represent independent observations.

The primary uniform composite included the four domains applicable to all 100 scenarios. Median scores were 4.00 (IQR 3.00–4.00) for ChatGPT, 4.56 (IQR 4.00–5.00) for Claude, 4.25 (IQR 3.34–5.00) for DeepSeek, and 4.25 (IQR 3.00–5.00) for Qwen. In the 51 common-proceed scenarios, the secondary five-domain composite medians were 3.40 (IQR 2.60–3.40), 5.00 (IQR 4.30–5.00), 5.00 (IQR 4.10–5.00), and 4.40 (IQR 3.40–5.00), respectively. Across all 3806 individual domain ratings, 92.7% were at the scale extremes (scores 1 or 5), whereas scores 2–4 accounted for 7.3%. Domain-level results are therefore presented using medians, IQRs, full score distributions, and the proportion of ratings judged appropriate (scores 4–5) with 95% bootstrap confidence intervals (Table 2 and Figure 1).

Fully appropriate medication-discontinuation ratings (score 5) were 91.0% for ChatGPT, 97.0% for Claude, 91.0% for DeepSeek, and 99.0% for Qwen. Cochran’s Q was significant (14.57, *p* = 0.002), but no pairwise exact McNemar comparison remained significant after Bonferroni correction. Accordingly, the omnibus result was not interpreted as evidence of a specific between-model difference in medication-discontinuation performance.

Median discontinuation-timing ratings were 5 (IQR 1–5) for ChatGPT, 5 (IQR 1–5) for Claude, 5 (IQR 4–5) for DeepSeek, and 5 (IQR 5–5) for Qwen. Proceed/postpone medians were 5 (IQR 5–5), 5 (IQR 5–5), 5 (IQR 1–5), and 5 (IQR 1–5), respectively. Anesthesia-type and clinical-rationale ratings were more variable (Table 2). Appropriate ratings (scores 4–5) ranged from 91.0% to 99.0% for medication discontinuation, 56.0% to 94.5% for discontinuation timing, 66.5% to 84.5% for the surgical decision, 39.7% to 89.6% for anesthesia type, and 32.0% to 85.0% for clinical rationale across models (Table 2).

### 3.3. Domain-Level Between-Model Comparisons

Significant between-model differences were observed for discontinuation timing (Friedman χ^2^ = 40.53, *p* < 0.001), the decision to proceed with or postpone surgery (χ^2^ = 20.70, *p* < 0.001), and clinical rationale (χ^2^ = 71.45, *p* < 0.001). For discontinuation timing, Bonferroni-adjusted pairwise differences were significant for ChatGPT–DeepSeek (*p* = 0.002), ChatGPT–Qwen (*p* < 0.001), Claude–Qwen (*p* < 0.001), and DeepSeek–Qwen (*p* < 0.001), but not for ChatGPT–Claude (*p* = 1.000) or Claude–DeepSeek (*p* = 0.755).

For the surgical-decision domain, significant differences were observed for ChatGPT–DeepSeek (*p* = 0.022) and Claude–DeepSeek (*p* = 0.021), whereas ChatGPT–Qwen (*p* = 0.053), Claude–Qwen (*p* = 0.059), ChatGPT–Claude (*p* = 1.000), and DeepSeek–Qwen (*p* = 1.000) were not significant. For clinical rationale, all pairwise comparisons were significant after adjustment except ChatGPT–Qwen (*p* = 1.000): ChatGPT–Claude (*p* < 0.001), ChatGPT–DeepSeek (*p* < 0.001), Claude–DeepSeek (*p* = 0.023), Claude–Qwen (*p* < 0.001), and DeepSeek–Qwen (*p* < 0.001).

### 3.4. Between-Model Comparisons

Using the uniform four-domain composite across all 100 scenarios, overall expert-rated performance differed significantly across models (Friedman χ^2^ = 28.75, *p* < 0.001; Kendall’s W = 0.096). After Bonferroni adjustment, significant differences were observed between ChatGPT and Claude (*p* < 0.001), ChatGPT and DeepSeek (*p* = 0.021), and Claude and Qwen (*p* = 0.011). Differences between ChatGPT and Qwen (*p* = 0.112), Claude and DeepSeek (*p* = 0.452), and DeepSeek and Qwen (*p* = 1.000) were not statistically significant (Table 3).

Leave-one-domain-out sensitivity analyses showed that the magnitude of between-model differences varied according to the domain excluded from the primary composite. Excluding medication discontinuation, discontinuation timing, or the surgical-decision domain did not attenuate the overall between-model separation (Friedman χ^2^ = 30.46, 49.69, and 39.26; Kendall’s W = 0.102, 0.166, and 0.131, respectively; all *p* < 0.001). In contrast, exclusion of clinical rationale substantially reduced the overall between-model difference (Friedman χ^2^ = 9.05, *p* = 0.029; Kendall’s W = 0.030). In this three-domain analysis, only the ChatGPT–Qwen (adjusted *p* = 0.031) and DeepSeek–Qwen (adjusted *p* = 0.001) comparisons remained statistically significant. These findings indicate that clinical-rationale ratings contributed substantially to the separation observed in the primary composite.

In the secondary analysis restricted to the 51 scenarios in which all four models recommended proceeding, the five-domain composite also differed across models (Friedman χ^2^ = 55.13, *p* < 0.001; Kendall’s W = 0.360). All 51 scenarios in this common-proceed subset were classified as standard scenarios; none of the 48 grey-zone scenarios were included. DeepSeek and Qwen each recommended proceeding in 53 scenarios, and their proceed sets overlapped in 51 cases. Pairwise differences were significant for ChatGPT versus Claude, DeepSeek, and Qwen, and for Claude versus Qwen (all adjusted *p* < 0.001), whereas Claude–DeepSeek (*p* = 1.000) and DeepSeek–Qwen (*p* = 0.485) were not significant (Table 3).

### 3.5. Clinical Applicability

Clinical applicability differed across models for both evaluators (Evaluator 1: Cochran’s Q = 16.24, *p* = 0.001; Evaluator 2: Cochran’s Q = 39.50, *p* < 0.001). Positive ratings for ChatGPT, Claude, DeepSeek, and Qwen were 25.0%, 52.0%, 46.0%, and 45.0% for Evaluator 1 and 20.0%, 54.0%, 59.0%, and 52.0% for Evaluator 2; pooled descriptive rates were 22.5%, 53.0%, 52.5%, and 48.5%, respectively. These pooled proportions represent descriptive averages of two independent evaluator ratings rather than adjudicated consensus estimates. Evaluator-specific positive applicability rates were 25% and 20% for ChatGPT, 52% and 54% for Claude, 46% and 59% for DeepSeek, and 45% and 52% for Qwen; the largest between-rater difference was observed for DeepSeek. After Bonferroni adjustment, Evaluator 1 found a significant difference only for ChatGPT versus Claude (*p* = 0.002); Evaluator 2 found ChatGPT differed from Claude, DeepSeek, and Qwen (all *p* < 0.001), with no differences among the latter three.

### 3.6. Surgical Postponement Behaviour

Postponement behaviour varied substantially across models: Claude did not recommend postponement in any scenario, ChatGPT did so in 3%, and DeepSeek and Qwen in 47% each. Postponement frequency alone was not treated as a measure of decision quality. In the proceed/postpone domain, completely inappropriate ratings (score 1) were observed in 12.0% of ratings for ChatGPT, 12.0% for Claude, 30.5% for DeepSeek, and 28.0% for Qwen. The overall domain comparison was significant (Friedman χ^2^ = 20.70, *p* < 0.001). After Bonferroni adjustment, DeepSeek differed significantly from ChatGPT (*p* = 0.022) and Claude (*p* = 0.021), whereas the corresponding comparisons for Qwen with ChatGPT (*p* = 0.053) and Claude (*p* = 0.059) did not reach statistical significance. Most postponements occurred in grey-zone scenarios.

### 3.7. Anesthesia Type

Anesthesia type selection was evaluated only in cases where surgery was recommended to proceed. Anesthesia ratings were first summarized within each model’s own proceed cases. The primary comparison used the 51 scenarios in which all four models proceeded. Median ratings were 1 (IQR 1–5) for ChatGPT, 5 (IQR 5–5) for Claude, 5 (IQR 5–5) for DeepSeek, and 5 (IQR 1–5) for Qwen; Friedman χ^2^ = 31.92, *p* < 0.001. Bonferroni-adjusted Wilcoxon tests were significant for ChatGPT versus Claude, DeepSeek, and Qwen (all *p* < 0.001), Claude versus Qwen (*p* = 0.003), and DeepSeek versus Qwen (*p* = 0.006), but not Claude versus DeepSeek (*p* = 1.000) (Table 4).

### 3.8. Exploratory Characterization of Score-1 Outputs

Exploratory review of responses associated with score-1 ratings identified several recurring patterns. Among the 110 individual ChatGPT anesthesia-type ratings scored 1, 104 (94.5%) were associated with responses recommending spinal anesthesia. Among the 127 ChatGPT clinical-rationale ratings scored 1, 109 (85.8%) were associated with a recurring generic statement that coagulation parameters and platelet count were appropriate and that neuraxial safety was ensured. In the proceed/postpone domain, 22 of 24 (91.7%) ChatGPT score-1 ratings and all 24 of 24 (100%) Claude score-1 ratings were associated with recommendations to proceed with surgery. In contrast, 58 of 61 (95.1%) DeepSeek and 55 of 56 (98.2%) Qwen score-1 ratings were associated with postponement recommendations. For Claude, 16 anesthesia-type ratings received a score of 1 across eight scenarios; 14 of these 16 ratings (87.5%), corresponding to seven of the eight scenarios, were associated with spinal anesthesia recommendations. These eight scenarios were outside the 51-scenario common-proceed subset. These findings are descriptive and non-adjudicated and should not be interpreted as a formal classification of safety errors. Representative examples are provided in the Supplementary File S12.

### 3.9. Grey-Zone Analysis

Using the uniform four-domain composite, median scores in grey-zone versus standard scenarios were 4.00 (IQR 3.00–4.81) versus 4.00 (IQR 3.00–4.00) for ChatGPT; 4.00 (IQR 3.75–4.56) versus 5.00 (IQR 4.50–5.00) for Claude; 3.50 (IQR 3.22–4.41) versus 5.00 (IQR 4.22–5.00) for DeepSeek; and 3.00 (IQR 3.00–4.91) versus 4.25 (IQR 4.00–5.00) for Qwen. After Bonferroni adjustment, grey-zone scores were lower than standard-scenario scores for Claude (adjusted *p* < 0.001), DeepSeek (adjusted *p* < 0.001), and Qwen (adjusted *p* = 0.004), whereas ChatGPT showed no significant difference (adjusted *p* = 1.000) (Table 5).

The leave-one-domain-out sensitivity analysis is summarized in Table 6.

## 4. Discussion

This study compared expert-rated LLM performance in standardized perioperative antithrombotic scenarios. ESAIC/ESRA 2022 provided a common framework for neuraxial safety, while broader perioperative decisions tested each model’s independent clinical reasoning. The findings indicate that there are notable differences among models not only in overall performance but also across specific clinical decision domains. This suggests that although LLMs hold promise in clinical decision support, their performance remains context-sensitive and model-dependent. The leave-one-domain-out sensitivity analysis further showed that the primary composite was not equally influenced by its constituent domains. Removing medication discontinuation, discontinuation timing, or the surgical-decision domain did not diminish between-model separation, whereas removal of clinical rationale markedly reduced the overall effect size. This indicates that clinical-rationale performance contributed substantially to the observed composite differences. Accordingly, the primary composite should be interpreted as a summary of performance across the assessed decision domains rather than as a general measure of clinical reasoning ability. The direction of several between-model differences was similar across the primary and secondary composite specifications, although the magnitude of separation increased in the secondary analysis. Importantly, the 51-scenario common-proceed subset consisted entirely of standard scenarios and was determined largely by the proceed decisions of DeepSeek and Qwen. At the case level, DeepSeek and Qwen each recommended proceeding in 53 scenarios, with 51 of these scenarios overlapping, indicating a high degree of concordance in their proceed/postpone behaviour. The secondary analysis should therefore be interpreted as a within-subset description rather than as independent confirmation of the primary model ordering. However, some pairwise differences varied between these specifications, suggesting that fine-grained model rankings should be interpreted cautiously. This caution is further reinforced by the single-query design. Because each scenario was sampled only once from each model, within-model stochastic variability was not measured, and differences among closely ranked models cannot be clearly separated from variability associated with a single generated response.

Claude and DeepSeek generally received higher expert ratings, whereas ChatGPT scored lower in discontinuation timing, anesthesia-type selection, and clinical justification. Similarly, a study based on real patient data reported that AI applications showed variable agreement with expert decisions in anesthesia selection and had limitations in comprehensively evaluating clinical exceptions and individual patient characteristics [9]. The literature also demonstrates significant variability among LLMs in terms of clinical accuracy and decision quality [10,11]. In line with this, it has been reported that AI-generated clinical scenarios may differ in content accuracy, clinical consistency and educational value [12]. Another study showed that AI-based models can achieve performance comparable to experts in certain standardized tasks, particularly in structural organization and linguistic fluency; however, human experts remain superior in critical aspects such as source transparency and factual accuracy [13] (co-authored by two non-author contributors to the present study, K.T. and H.Y.A.). Taken together, these findings suggest that although LLMs have strong capacity for accessing medical knowledge, their ability to organize this information contextually and integrate it into clinical reasoning varies across models.

Findings related to anesthesia type selection similarly revealed model-specific differences. Because models differed in which cases they chose to proceed, between-model interpretation focused on the 51 scenarios in which all four proceeded. This common subset reduced self-selection from model-specific proceed sets; findings therefore apply to these shared scenarios rather than differently selected case sets.

The divergence in model performance also varied according to the type of decision. Because ratings were strongly concentrated at the scale extremes, domain-level interpretation was complemented by appropriate-rating proportions rather than relying on mean scores as indicators of clinical quality. In our study, models performed well in more algorithmic and guideline-based areas such as drug discontinuation decisions, whereas performance declined in more nuanced domains requiring contextual reasoning, such as clinical justification. The exploratory review of score-1 ratings provided additional context for these domain-level differences. ChatGPT’s lowest anesthesia-type ratings were predominantly associated with spinal anesthesia recommendations, and a similar pattern was observed among Claude’s anesthesia-type score-1 ratings outside the common-proceed subset. Low-rated surgical decisions occurred in both directions: score-1 proceed/postpone ratings for DeepSeek and Qwen were predominantly associated with postponement, whereas those for ChatGPT and Claude were predominantly associated with recommendations to proceed. This directional contrast illustrates that low expert ratings were not confined to a single decision tendency and that postponement frequency alone does not capture decision quality. These patterns describe the types of responses that received the lowest ratings rather than establishing specific mechanisms of error or clinical harm. Because the characterization was performed post hoc and without independent adjudication, the findings should be considered hypothesis-generating. This suggests that current LLMs are more effective in structured decision-making tasks but remain limited in complex, multidimensional clinical reasoning. Indeed, a study evaluating guideline-based neuraxial bleeding risk assessment demonstrated that GPT-3.5 performance was initially limited but improved significantly with structured and detailed prompts [14]. However, that study evaluated GPT-3.5 against ASRA guidance, whereas the present study used a different model set and reference framework, limiting direct comparability. Similarly, studies focusing on regional anesthesia decisions in anticoagulated trauma patients emphasize that AI-assisted approaches may support clinical decision-making but require careful interpretation [15]. Ruan et al. reported a different ranking in complex obstetric and geriatric anesthesia scenarios, with DeepSeek-R1 highest and Claude 3.5 Sonnet lowest, whereas Claude had the highest median primary composite score in the present study and did not differ significantly from DeepSeek. Differences in case mix, model versions, prompts, assessment domains, and evaluator panels suggest that model rankings are context-dependent rather than stable [16]. In contrast, Antiperovitch et al. evaluated seven LLMs using validated cardiovascular antithrombotic scenarios and benchmarked their performance against clinicians of different experience levels. Claude 3 Opus achieved the highest accuracy, correctly managing 85% of the scenarios. Although the clinical setting, model versions, and evaluation approach differed from those of the present study, this finding is broadly consistent with the comparatively strong performance observed for Claude in our analysis. Importantly, their use of an expert-defined reference standard and a clinician comparator allowed model performance to be interpreted in terms of scenario accuracy and human benchmarking [17]. These findings suggest that while LLMs may offer potential benefits in clinical decision-making, their reliable use requires careful consideration.

Clinical applicability analyses provide an important perspective for evaluating the real-world relevance of model outputs. The observation that models with higher scores also demonstrated higher applicability rates suggests a degree of alignment between theoretical accuracy and clinical suitability. However, the presence of recommendations that appear superficially correct but are not clinically applicable highlights the risks of using LLM outputs as direct clinical decisions. Indeed, it has been shown that LLMs may accept incorrect medical information presented within clinical texts, particularly when expressed in authoritative language [18]. This is consistent with studies emphasizing that AI systems should be positioned as clinical decision support tools and that issues such as data security, transparency and ethical responsibility must be carefully addressed for safe and effective implementation [19].

Postponement frequency alone should not be interpreted as a measure of decision quality; however, the appropriateness ratings provide additional context. DeepSeek and Qwen had higher proportions of completely inappropriate proceed/postpone ratings (30.5% and 28.0%, respectively) than ChatGPT and Claude (12.0% each). In paired domain-level comparisons, DeepSeek differed significantly from both ChatGPT and Claude after multiplicity adjustment, whereas the corresponding Qwen comparisons did not reach statistical significance. The exploratory characterization further showed that these low-rated decisions were not unidirectional: score-1 surgical-decision ratings were predominantly associated with postponement for DeepSeek and Qwen, but with proceeding for ChatGPT and Claude. This pattern reinforces that postponement frequency alone should not be interpreted as a quality measure; the appropriateness of the individual decision remains the clinically relevant outcome.

Using the uniform four-domain composite, grey-zone scores were significantly lower than standard-scenario scores for Claude, DeepSeek, and Qwen after Bonferroni adjustment, whereas no significant difference was observed for ChatGPT. This null finding should not be interpreted as evidence that ChatGPT was insensitive to grey-zone status, as the similar composite-score distributions across the two groups may have limited the ability of this analysis to detect a difference. Because grey-zone status was a pragmatic rather than validated classification, these exploratory findings should not be interpreted as definitive evidence of an effect of scenario complexity or as favouring any particular postponement strategy.

In conclusion, although large language models show considerable potential in complex clinical domains such as perioperative antithrombotic management, their current performance—particularly in grey-zone and high-risk scenarios does not support their use as independent decision-makers. When appropriately structured and used under expert supervision, these systems may serve as valuable tools for education and clinical decision support. Despite the increasing use of clinical decision support systems, clinicians continue to approach these technologies with caution. Indeed, a survey-based study reported that while many clinicians acknowledge the potential benefits of digital decision support tools, they emphasize that these systems should support rather than replace physician decision-making [20]. Therefore, expert oversight remains essential for the safe use of LLMs in clinical practice.

The study’s main contribution is a structured comparison of LLM-generated perioperative antithrombotic recommendations across five clinical domains, including an exploratory grey-zone analysis, using identical scenarios across models. Key strengths are the scenario-based design, concealed model identity, and generally strong inter-rater agreement. Inter-rater reliability was generally strong, although agreement was lower for DeepSeek in the anesthesia-type domain, underscoring the value of domain-specific rather than pooled reliability reporting. Furthermore, the comparison of different LLMs on identical clinical scenarios allows for direct assessment of model performance.

Several limitations should be noted. Initial scenario drafting with Gemini Pro may have introduced model-mediated framing bias despite expert review; future studies should test human-authored or mixed-origin case sets. The single-centre design and two-expert panel also limit external validity. Although inter-rater agreement was generally high, it reflects consistency within this panel rather than across institutions, particularly for grey-zone decisions; larger multicenter panels are needed. No independent prespecified reference standard was established, so scores represent expert-rated clinical appropriateness rather than objective guideline concordance. A reference-standard design with a human comparator, as used by Antiperovitch et al., would have allowed direct estimation of guideline-concordant accuracy and comparison with clinician performance; its absence in the present study limits interpretation to expert-rated clinical appropriateness [17]. The expert-rated appropriateness framework also did not distinguish the direction of an inappropriate recommendation. For example, an unnecessarily conservative postponement and a potentially insufficient antithrombotic interruption could both receive the same low score despite having different clinical implications. Accordingly, the primary endpoint should not be interpreted as distinguishing over-cautious from under-cautious decision-making. A prospective evaluation using prespecified, direction-specific error categories and an independently adjudicated guideline-concordance reference would be needed to address this distinction. No prespecified taxonomy was used for hallucinations, guideline deviations, critical errors, or potentially harmful recommendations. The post hoc characterization of score-1 responses was therefore limited to descriptive, non-adjudicated patterns and should not be interpreted as a validated safety analysis. Future studies should incorporate prespecified error definitions and independent adjudication to distinguish factual errors, guideline deviations, and potentially harmful recommendations more systematically. ESAIC/ESRA 2022 served as a common framework for neuraxial safety, but several broader perioperative decisions lay outside its direct scope. Models were tested through consumer web interfaces in February–March 2026; backend identifiers, temperature settings, and random seeds were unavailable. Although no external web search or tool use was intentionally invoked, account-level memory and personalization settings were not independently verified across interfaces; therefore, complete exclusion of account-level contextual effects cannot be confirmed. Each scenario was queried once, precluding assessment of within-model stochastic variability and test–retest reproducibility. Consequently, fine-grained differences among closely ranked models may partly reflect single-draw variability rather than stable performance differences. Future studies should use repeated sampling within controlled model versions or fixed API snapshots to assess the stability of model-level comparisons. Use of a single structured prompt further limits the generalizability of model rankings. In addition, instructing the models to respond from the perspective of an experienced anesthesiologist constituted a persona-based prompt-design choice that may have influenced response framing and recommendations. Future studies should assess the robustness of model performance across alternative prompt formulations and persona instructions. Although explicit model identifiers were removed and response order was independently randomized, blinding efficacy was not formally assessed. Given the recurring model-specific response patterns and marked differences in postponement behaviour observed across repeated assessments, model identity may have become increasingly inferable to the evaluators over the course of the study. Blinding should therefore be regarded as incomplete, and expectancy effects related to perceived model identity cannot be excluded as a source of bias in between-model comparisons. Grey-zone status was a pragmatic, nonvalidated designation, making subgroup findings exploratory. Finally, simulated scenarios may not fully reflect real clinical practice.

## 5. Conclusions

In this scenario-based expert evaluation, LLM performance varied across perioperative antithrombotic decision domains. While these findings underscore the emerging potential of LLMs as clinical decision support tools, they also highlight that, in their current state, these systems are not suitable for autonomous clinical decision-making. Ensuring patient safety requires that LLMs be used strictly under expert supervision and within a supportive, rather than substitutive, role. Future studies should test these findings across prompts, model versions, evaluator panels, and real-world settings.

## Figures and Tables

**Table 1:** Inter-rater agreement by model and assessment domain.

Assessment Dimension	ChatGPT	Claude	DeepSeek	Qwen
Assessment Dimension	Exact Agreement/uκ/wκ/ICC(2,1) (95% CI)	Exact Agreement/uκ/wκ/ICC(2,1) (95% CI)	Exact Agreement/uκ/wκ/ICC(2,1) (95% CI)	Exact Agreement/uκ/wκ/ICC(2,1) (95% CI)
Medication Discontinuation Decision	100%; uκ = 1.000; wκ = NA; ICC = 1.000 (1.000–1.000)	100%; uκ = 1.000; wκ = NA; ICC = 1.000 (1.000–1.000)	100%; uκ = 1.000; wκ = NA; ICC = 1.000 (1.000–1.000)	100%; uκ = 1.000; wκ = NA; ICC = 1.000 (1.000–1.000)
Discontinuation Timing	96.0%; uκ = 0.925; wκ = 0.991; ICC = 0.995 (0.989–0.999)	96.0%; uκ = 0.917; wκ = 0.994; ICC = 0.994 (0.988–0.999)	95.0%; uκ = 0.878; wκ = 0.984; ICC = 0.991 (0.980–0.998)	97.0%; uκ = 0.775; wκ = 0.963; ICC = 0.978 (0.909–1.000)
Decision to Proceed with Surgery	93.0%; uκ = 0.758; wκ = 0.935; ICC = 0.935 (0.851–0.985)	92.0%; uκ = 0.738; wκ = 0.932; ICC = 0.933 (0.848–0.984)	94.0%; uκ = 0.874; wκ = 0.963; ICC = 0.963 (0.917–0.996)	92.0%; uκ = 0.828; wκ = 0.930; ICC = 0.931 (0.852–0.985)
Anesthesia Type ^†^	95.9%; uκ = 0.922; wκ = 0.990; ICC = 0.990 (0.976–0.999)	91.0%; uκ = 0.710; wκ = 0.946; ICC = 0.946 (0.874–0.983)	79.2%; uκ = 0.393; wκ = 0.627; ICC = 0.631 (0.429–0.780)	96.2%; uκ = 0.924; wκ = 0.991; ICC = 0.995 (0.985–1.000)
Clinical Rationale	95.0%; uκ = 0.901; wκ = 0.988; ICC = 0.988 (0.972–0.998)	97.0%; uκ = 0.925; wκ = 0.970; ICC = 0.982 (0.944–1.000)	83.0%; uκ = 0.730; wκ = 0.852; ICC = 0.854 (0.762–0.923)	90.0%; uκ = 0.847; wκ = 0.942; ICC = 0.942 (0.896–0.978)
Clinical Applicability (binary)	95.0%; uκ = 0.857; wκ = NA; ICC = NA	88.0%; uκ = 0.759; wκ = NA; ICC = NA	85.0%; uκ = 0.704; wκ = NA; ICC = NA	93.0%; uκ = 0.861; wκ = NA; ICC = NA

uκ = unweighted Cohen’s kappa; wκ = quadratic-weighted Cohen’s kappa. Unweighted kappa was calculated for all domains; quadratic-weighted kappa was additionally reported for ordinal domains. Because medication discontinuation used only scores 1 and 5 with perfect inter-rater agreement, weighted kappa was not informative for that domain, and the ICC of 1.000 reflects this degenerate variance structure rather than a broadly distributed continuous measure of agreement. ICCs are two-way random-effects, absolute-agreement, single-measure ICC(2,1), with 95% CIs from 10,000 case-level bootstrap resamples. ^†^ Anesthesia-type agreement used evaluable cases only (ChatGPT *n* = 97; Claude *n* = 100; DeepSeek *n* = 53; Qwen *n* = 53). Across 1903 evaluable scenario-domain pairs, 12 (0.6%) were midpoint-straddling disagreements (one rater scored 1–2 and the other 4–5); one pair (0.05%) was an extreme 1-versus-5 disagreement. Clinical applicability was a binary yes/no item and was therefore summarized with exact agreement and unweighted Cohen’s kappa only; weighted kappa and ICC were not applicable. Applicability agreement used all 100 scenarios per model.

**Table 2:** Distribution of expert ratings across clinical decision domains.

Dimension	Statistic	ChatGPT	Claude	DeepSeek	Qwen
Medication Discontinuation Decision	Median (IQR)	5 (5–5)	5 (5–5)	5 (5–5)	5 (5–5)
	Score distribution 1/2/3/4/5, *n* (%)	18 (9.0)/0/0/0/182 (91.0)	6 (3.0)/0/0/0/194 (97.0)	18 (9.0)/0/0/0/182 (91.0)	2 (1.0)/0/0/0/198 (99.0)
	Appropriate ratings (4–5), *n*/*N* (%) [95% CI]	182/200 (91.0%) [85.0–96.0]	194/200 (97.0%) [93.0–100.0]	182/200 (91.0%) [85.0–96.0]	198/200 (99.0%) [97.0–100.0]
Discontinuation Timing	Median (IQR)	5 (1–5)	5 (1–5)	5 (4–5)	5 (5–5)
	Score distribution 1/2/3/4/5, *n* (%)	84 (42.0)/0/4 (2.0)/4 (2.0)/108 (54.0)	65 (32.5)/1 (0.5)/3 (1.5)/3 (1.5)/128 (64.0)	42 (21.0)/0/5 (2.5)/5 (2.5)/148 (74.0)	8 (4.0)/0/3 (1.5)/3 (1.5)/186 (93.0)
	Appropriate ratings (4–5), *n*/*N* (%) [95% CI]	112/200 (56.0%) [46.5–65.5]	131/200 (65.5%) [56.0–74.5]	153/200 (76.5%) [68.5–84.0]	189/200 (94.5%) [90.0–98.0]
Decision to Proceed with Surgery	Median (IQR)	5 (5–5)	5 (5–5)	5 (1–5)	5 (1–5)
	Score distribution 1/2/3/4/5, *n* (%)	24 (12.0)/4 (2.0)/3 (1.5)/2 (1.0)/167 (83.5)	24 (12.0)/4 (2.0)/4 (2.0)/3 (1.5)/165 (82.5)	61 (30.5)/3 (1.5)/3 (1.5)/2 (1.0)/131 (65.5)	56 (28.0)/3 (1.5)/4 (2.0)/2 (1.0)/135 (67.5)
	Appropriate ratings (4–5), *n*/*N* (%) [95% CI]	169/200 (84.5%) [77.5–91.0]	168/200 (84.0%) [77.0–90.5]	133/200 (66.5%) [57.5–75.0]	137/200 (68.5%) [59.5–77.0]
Anesthesia Type ^‡^	Median (IQR)	1 (1–5)	5 (5–5)	5 (5–5)	5 (1–5)
	Score distribution 1/2/3/4/5, *n* (%)	110 (56.7)/1 (0.5)/6 (3.1)/2 (1.0)/75 (38.7)	16 (8.0)/3 (1.5)/8 (4.0)/8 (4.0)/165 (82.5)	0/0/11 (10.4)/9 (8.5)/86 (81.1)	36 (34.0)/0/2 (1.9)/2 (1.9)/66 (62.3)
	Appropriate ratings (4–5), *n*/*N* (%) [95% CI]	77/194 (39.7%) [30.4–49.0]	173/200 (86.5%) [80.0–92.5]	95/106 (89.6%) [84.0–94.3]	68/106 (64.2%) [50.9–76.4]
Clinical Rationale	Median (IQR)	1 (1–5)	5 (5–5)	5 (3–5)	2 (1–5)
	Score distribution 1/2/3/4/5, *n* (%)	127 (63.5)/5 (2.5)/4 (2.0)/3 (1.5)/61 (30.5)	22 (11.0)/0/8 (4.0)/18 (9.0)/152 (76.0)	17 (8.5)/15 (7.5)/52 (26.0)/8 (4.0)/108 (54.0)	94 (47.0)/34 (17.0)/6 (3.0)/4 (2.0)/62 (31.0)
	Appropriate ratings (4–5), *n*/*N* (%) [95% CI]	64/200 (32.0%) [23.0–41.5]	170/200 (85.0%) [78.0–91.5]	116/200 (58.0%) [49.0–67.0]	66/200 (33.0%) [24.5–42.0]
PRIMARY FOUR-DOMAIN COMPOSITE ^††^	Median (IQR)	4.00 (3.00–4.00)	4.56 (4.00–5.00)	4.25 (3.34–5.00)	4.25 (3.00–5.00)
SECONDARY FIVE-DOMAIN COMPOSITE ^‡‡^	Median (IQR)	3.40 (2.60–3.40)	5.00 (4.30–5.00)	5.00 (4.10–5.00)	4.40 (3.40–5.00)

Domain distributions are descriptive counts from all individual evaluator ratings (two ratings per evaluable case). Across all 3806 individual domain ratings, 92.7% were at the scale extremes (scores 1 or 5), while scores 2–4 accounted for 7.3%. Appropriate ratings were defined as scores 4–5. Percentages are based on individual evaluator ratings, whereas 95% confidence intervals were obtained from 10,000 case-level bootstrap resamples that preserved the paired rater ratings within each scenario. ^‡^ Anesthesia type was scored only when the model recommended proceeding (ChatGPT *n* = 97; Claude *n* = 100; DeepSeek *n* = 53; Qwen *n* = 53). ^††^ The primary four-domain composite uses medication discontinuation, discontinuation timing, surgical decision, and clinical rationale across all 100 scenarios. ^‡‡^ The secondary five-domain composite includes anesthesia type and is restricted to the 51 scenarios in which all four models recommended proceeding. Composite scores are reported as median (IQR).

**Table 3:** Pairwise comparisons of primary and secondary composite scores.

Comparison	W	Raw *p*	Bonferroni-Adjusted *p*	Significant	Effect Size (r)
A. Primary uniform four-domain composite (*n* = 100)					
ChatGPT vs. Claude	629.5	<0.001	<0.001	Yes	0.33
ChatGPT vs. DeepSeek	1531.5	0.0035	0.0209	Yes	0.21
ChatGPT vs. Qwen	1163.0	0.0187	0.1123	No	0.17
Claude vs. DeepSeek	1120.5	0.0754	0.4521	No	0.13
Claude vs. Qwen	1120.0	0.0018	0.0109	Yes	0.22
DeepSeek vs. Qwen	1284.0	0.2668	1.000	No	0.08
B. Secondary five-domain composite in the common-proceed subset (*n* = 51)					
ChatGPT vs. Claude	29.0	<0.001	<0.001	Yes	0.58
ChatGPT vs. DeepSeek	85.0	<0.001	<0.001	Yes	0.53
ChatGPT vs. Qwen	46.0	<0.001	<0.001	Yes	0.49
Claude vs. DeepSeek	179.5	0.4088	1.000	No	0.08
Claude vs. Qwen	127.5	<0.001	<0.001	Yes	0.40
DeepSeek vs. Qwen	364.0	0.0808	0.4847	No	0.17

Table 3 note: Panel A shows the primary uniform four-domain composite across all 100 scenarios (Friedman χ^2^ = 28.75, *p* < 0.001; Kendall’s W = 0.096). Panel B shows the secondary five-domain composite restricted to the 51 common-proceed scenarios; all 51 were standard scenarios and none of the 48 grey-zone scenarios were included (Friedman χ^2^ = 55.13, *p* < 0.001; Kendall’s W = 0.360). Pairwise *p* values are Bonferroni-adjusted across six comparisons and compared with α = 0.05. Effect size was calculated using Rosenthal’s convention as r = |Z|/√N_total, where N_total is the total number of observations contributing to the paired comparison (2 × the number of paired scenarios): N_total = 200 for Panel A and N_total = 102 for Panel B.

**Table 4:** Primary between-model anesthesia comparison in the common-proceed subset.

Model	Scenarios, *n*	Median (IQR)	Score = 5 *n* (%)	Score = 1 *n* (%)	Primary Between-Model Analysis
ChatGPT	51	1 (1–5)	32/102 (31.4%)	68/102 (66.7%)	Friedman χ^2^ = 31.92; *p* < 0.001
Claude	51	5 (5–5)	87/102 (85.3%)	0/102 (0.0%)	
DeepSeek	51	5 (5–5)	82/102 (80.4%)	0/102 (0.0%)	
Qwen	51	5 (1–5)	66/102 (64.7%)	32/102 (31.4%)	

Primary analysis was restricted to the 51 scenarios in which all four models recommended proceeding with surgery. All 51 were standard scenarios; none of the 48 grey-zone scenarios were included. Median (IQR), Score = 5 *n* (%), and Score = 1 *n* (%) are descriptive summaries based on the 102 individual evaluator ratings per model (two ratings for each of 51 scenarios). For inferential testing, the two evaluator ratings were averaged within each scenario to obtain one paired anesthesia-type score per model (*n* = 51 scenario-level values). Friedman χ^2^(3) = 31.92, *p* < 0.001. Bonferroni-adjusted pairwise Wilcoxon *p* values: ChatGPT vs. Claude < 0.001; ChatGPT vs. DeepSeek < 0.001; ChatGPT vs. Qwen < 0.001; Claude vs. DeepSeek = 1.000; Claude vs. Qwen = 0.003; DeepSeek vs. Qwen = 0.006.

**Table 5:** Comparison of primary four-domain composite scores between grey-zone and standard scenarios.

Model	Group	*n*	Mean ± SD (95% CI)	Median (IQR)	U	Raw *p*	Bonferroni-Adjusted *p*	Effect Size (r)
ChatGPT	Grey-zone	48	3.72 ± 1.09 (3.41–4.01)	4.00 (3.00–4.81)	1334	0.544	1.000	0.06
ChatGPT	Standard	52	3.61 ± 0.87 (3.38–3.84)	4.00 (3.00–4.00)				
Claude	Grey-zone	48	3.93 ± 0.85 (3.69–4.17)	4.00 (3.75–4.56)	571	<0.001	<0.001	0.49
Claude	Standard	52	4.69 ± 0.49 (4.55–4.81)	5.00 (4.50–5.00)				
DeepSeek	Grey-zone	48	3.65 ± 0.92 (3.40–3.92)	3.50 (3.22–4.41)	660	<0.001	<0.001	0.42
DeepSeek	Standard	52	4.46 ± 0.83 (4.22–4.68)	5.00 (4.22–5.00)				
Qwen	Grey-zone	48	3.67 ± 0.98 (3.40–3.95)	3.00 (3.00–4.91)	779	0.001	0.004	0.33
Qwen	Standard	52	4.33 ± 0.55 (4.18–4.47)	4.25 (4.00–5.00)				

This exploratory analysis compares the uniform four-domain primary composite between 48 grey-zone and 52 standard scenarios. Values are presented as mean ± SD with 95% bootstrap confidence intervals for the group mean and as median (IQR). Confidence intervals were obtained from 10,000 bootstrap resamples. Mann–Whitney U tests were performed separately for each model, and *p* values were adjusted for four model-specific comparisons using the Bonferroni method. Effect size was calculated as r = |Z|/√N using the total number of scenarios in the two groups (*N* = 100). Adjusted *p* < 0.05 was considered statistically significant. Grey-zone status was recorded during initial case-set generation before model querying and expert rating; no separate rule-based classification algorithm was applied. CI: confidence interval; IQR: interquartile range.

**Table 6:** Leave-one-domain-out sensitivity analysis of the primary four-domain composite.

Domain Excluded	Friedman χ^2^	*p*	Kendall’s W	Significant Bonferroni-Adjusted Pairwise Comparisons
Medication discontinuation	30.46	<0.001	0.102	Claude > ChatGPT (*p* < 0.001); DeepSeek > ChatGPT (*p* = 0.017); Claude > Qwen (*p* = 0.009)
Discontinuation timing	49.69	<0.001	0.166	Claude > ChatGPT (*p* < 0.001); Claude > DeepSeek (*p* = 0.003); Claude > Qwen (*p* < 0.001); DeepSeek > Qwen (*p* = 0.012)
Surgical decision	39.26	<0.001	0.131	Claude > ChatGPT (*p* < 0.001); DeepSeek > ChatGPT (*p* < 0.001); Qwen > ChatGPT (*p* < 0.001)
Clinical rationale	9.05	0.029	0.030	Qwen > ChatGPT (*p* = 0.031); Qwen > DeepSeek (*p* = 0.001)

Each sensitivity composite was calculated across all 100 scenarios after sequentially excluding one of the four domains included in the primary composite. Omnibus comparisons used the Friedman test, with Kendall’s W as the effect-size measure. Pairwise Wilcoxon signed-rank tests were Bonferroni-adjusted across six model-to-model comparisons; only statistically significant adjusted comparisons are shown. The direction symbol indicates the model with higher paired composite scores. Anesthesia type was not part of the primary four-domain composite and was therefore not included in this leave-one-domain-out analysis.

## Data Availability

The original contributions presented in this study are included in the article/Supplementary Materials. Further inquiries can be directed to the corresponding author.

## Supplementary Materials

The following supporting information can be downloaded at: https://www.mdpi.com/article/10.3390/diagnostics16172848/s1, File S1, Standardized Master Prompt; File S2, Clinical Scenarios; Files S3–S6, Raw Model Responses; Files S7–S10, Item-Level Expert Ratings; File S11, Completed TRIPOD-LLM Checklist; File S12, Representative Patterns Among Score-1 Model Outputs.

## Abbreviations

LLMs	Large language models
AI	Artificial intelligence
ESAIC	European Society of Anaesthesiology and Intensive Care
ESRA	European Society of Regional Anesthesia and Pain Therapy
INR	International normalized ratio
ICC	Intraclass correlation coefficients
