# Supplementary material for: Large Language Models in Perioperative Antithrombotic Management: A Blinded Scenario-Based Expert Evaluation
Source: Diagnostics (Basel). 2026 Sep 4;16(17):2848. doi: 10.3390/diagnostics16172848 (PMC13564537; doi:10.3390/diagnostics16172848)
Supplement: Supplementary file 1 [file diagnostics-16-02848-s001.zip › File S11 TRIPOD LLM Checklist.pdf]

TRIPOD-LLM Reporting Checklist

Large Language Models in Perioperative Antithrombotic Management: A Blinded Scenario-Based Expert Evaluation

Checklist basis: TRIPOD-LLM (Gallifant et al., Nature Medicine 2025). This author-prepared checklist paraphrases the reporting items and applies the modules most relevant to an LLM evaluation in a healthcare decision-support setting with long-form, scenario-based responses.

Status terms: "Reported" indicates that the requested information is present in the manuscript or supplementary material; "N/A" indicates that the item does not apply to this study design; "Not available/not separately reported" is used where the information was unavailable or not explicitly stated.

Section	Item	Paraphrased reporting requirement	Study-specific completion/status	Location
Title	1	State that the study evaluates an LLM and identify the clinical task, target population/context, and outcome.	Partly reported. Title identifies LLM evaluation and perioperative antithrombotic management; clinical context and outcomes are detailed in the abstract/introduction.	p. 1, lines 1–2; pp. 2–5, lines 18–97
Abstract	2	Provide a structured abstract covering the essential TRIPOD-LLM elements.	Reported: design, 100 scenarios, models, expert evaluation, principal statistics/effect sizes, and conclusion are summarized.	pp. 2–3, lines 18–49
Introduction	3a	Explain the healthcare use case and why LLM evaluation is needed, in relation to prior work.	Reported.	pp. 3–4, lines 53–82
Introduction	3b	Describe the target clinical context, intended users, and intended role of the LLM in care.	Reported as simulated perioperative decision support/education rather than autonomous clinical use.	p. 5, lines 83–97; p. 19, lines 378–384
Introduction	4	State the study objectives and whether the work concerns development, tuning, or evaluation.	Reported as comparative LLM evaluation.	p. 5, lines 83–93
Methods – Data	5a	Describe the source and rationale for the evaluation data/case set.	Reported: 100 synthetic elective surgical scenarios developed for perioperative antithrombotic management.	pp. 5–6, lines 99–123
Methods – Data	5b	Describe the important data elements and their distribution/coverage.	Reported for antithrombotic class, indication, surgical context, renal/laboratory profiles, comorbidity, and grey-zone status; full cases supplied separately.	pp. 5–6, lines 99–121; Supplementary File S2
Methods – Data	5c	Report the relevant dates for source/evaluation text when meaningful.	Synthetic scenarios do not have source-document dates. Model querying period is reported.	p. 7, lines 125–135
Methods – Data	5d	Describe preprocessing and quality-control procedures applied to the evaluation data.	Reported: AI-assisted drafts underwent independent expert review/refinement before querying.	p. 6, lines 111–115; lines 116–121
Methods – Data	5e	Explain handling of missing/imbalanced data or omitted data.	No missing scenario data are reported. Anesthesia scores were structurally unavailable when surgery was postponed and were handled conditionally in analysis.	p. 9, lines 177–183; p. 10, lines 193–198
Methods – Analytical methods	6a	Report model name/version and, when available, last training date.	Model/interface labels and querying period are reported. Last training dates are not reported in the manuscript.	p. 7, lines 125–135
Methods – Analytical methods	6b	Describe LLM development/fine-tuning/alignment if performed.	Not applicable: no model development, fine-tuning, or alignment was performed by the authors.	N/A
Methods – Analytical methods	6c	Describe how text was generated, including prompt procedure and available inference settings.	Reported; full standardized prompt is provided. Backend identifiers, temperature, and random seeds were unavailable.	pp. 7–8, lines 125–155; Supplementary File S1
Methods – Analytical methods	6d	Specify the model output format and any post-processing.	Reported: structured clinical recommendations were requested; outputs were retained in original form and only explicit model identifiers were removed before rating.	p. 8, lines 146–157
Methods – Analytical methods	6e	If classification is used, explain class definitions, probabilities, and thresholds.	Not applicable: the models were not evaluated as probabilistic classifiers.	N/A
Methods – LLM output	7a	Report metrics used to judge generative output quality and the reference used for comparison.	Reported as expert-rated clinical appropriateness across five domains plus clinical applicability; no independent gold standard was claimed.	pp. 8–9, lines 158–183; p. 18, lines 361–366
Methods – LLM output	7b	Explain why the selected metrics are clinically relevant and their relationship to human evaluation.	Reported through expert domain ratings and the separate question on whether the recommendation would be applied clinically.	p. 9, lines 168–183; p. 13, lines 249–256
Methods – LLM output	7c	Define outcomes, inference timing, and evaluation metrics.	Reported, including February–March 2026 querying period and statistical evaluation framework.	p. 7, lines 125–135; pp. 8–11, lines 158–221
Methods – LLM output	7d	For subjective outcomes, report assessor expertise/instructions and agreement.	Reported for two anesthesiologists with ≥10 years' experience, blinded independent rating, pilot familiarization, and domain-specific agreement statistics. Detailed assessor demographics are not reported.	pp. 9–12, lines 173–227
Methods – LLM output	7e	Explain comparisons with other LLMs, humans, benchmarks, or standards.	Reported: identical scenarios were used across four LLMs; expert ratings provided the evaluation framework.	pp. 7–11, lines 125–221
Methods – Annotation	8a	Describe labeling/scoring instructions and anchor definitions when human annotation is used.	Reported through the five-domain 1–5 scoring framework and clinical-applicability question.	pp. 8–9, lines 158–172; Supplementary Files S7–S10
Methods – Annotation	8b	Report the number of annotators, duplicate annotation, and inter-annotator agreement.	Reported: two independent evaluators rated the responses; domain-specific exact agreement, kappa, and	pp. 9–12, lines 173–227

			ICC are reported.	
Methods – Annotation	8c	Describe annotator background and experience.	Reported.	p. 9, lines 173–176
Methods – Prompting	9a	Describe how prompts were designed, curated, selected, and applied.	Reported as one standardized prompt applied in new chat sessions without regeneration or extra prompting; no formal prompt-optimization experiment was performed.	p. 7, lines 136–143; Supplementary File S1
Methods – Prompting	9b	Describe the data/context used to construct the prompts.	Reported: clinical scenario variables and ESAIC/ESRA 2022 neuraxial-safety framework informed the task.	pp. 6–8, lines 105–155
Methods – Summarization	10	Describe preprocessing before summarization, if summarization is the task.	Not applicable.	N/A
Methods – Instruction tuning/alignment	11	If the authors performed instruction tuning/alignment, describe instructions, data, interface, and evaluators.	Not applicable: no tuning/alignment was performed.	N/A
Methods – Compute	12	Report compute requirements or reasonable proxies when available.	Compute and inference-time metrics were not measured or reported; the study used consumer web interfaces rather than APIs.	p. 7, lines 125–135
Methods – Ethics	13	Report ethics approval/waiver and consent when applicable.	Reported: no real patient or identifiable data were used; institutional approval was not required.	p. 6, lines 122–123; p. 20, lines 407–408
Open science	14a	Report funding and funder role.	Reported: no financial support.	p. 20, line 403
Open science	14b	Report conflicts of interest/financial disclosures.	Reported.	p. 20, line 409
Open science	14c	State where a protocol is available, or state that none was prepared.	Protocol availability is not reported in the manuscript.	Not reported
Open science	14d	Provide registration details or state that the study was not registered.	The manuscript states that a clinical-trial number is not applicable; the study is a simulation-based evaluation rather than a clinical trial.	p. 20, line 402
Open science	14e	Report availability of study data.	Reported: prompt, scenarios, model responses, and item-level ratings are supplied as Supplementary Files S1–S10.	p. 20, lines 404–406
Open science	14f	Report availability of code needed to reproduce the analyses.	No analysis code is provided; analyses were performed in IBM SPSS Statistics 29.0.	p. 10, lines 190–192
Patient/public involvement	15	Describe patient/public involvement or state that there was none.	Patient/public involvement is not reported. The study used synthetic scenarios and no patient data.	pp. 5–6, lines 94–123
Results – Participants/data flow	16a	For patient/EHR studies, show flow of participants/documents through the study.	Not applicable: no patient/EHR data were used.	N/A
Results – Participants/data flow	16b	For patient/EHR studies, report participant/data-source characteristics and sample sizes.	Not applicable: no patient/EHR data were used.	N/A
Results – Participants/data flow	16c	For clinical-outcome evaluations, compare important variables across development/evaluation data when relevant.	Not applicable: no clinical outcomes or development/evaluation patient cohorts were used.	N/A
Results – Participants/data flow	16d	For patient/EHR analyses, report participant and outcome-event counts for each analysis.	Not applicable.	N/A
Results – Performance	17	Report model performance using the prespecified metrics and/or human evaluation.	Reported across inter-rater agreement, domain distributions, overall comparisons, clinical applicability, anesthesia, and grey-zone analyses.	pp. 11–14, lines 222–276
Results – Updating	18	If the LLM was updated by the authors, report the update and post-update performance.	Not applicable: no model updating was performed.	N/A
Discussion	19a	Interpret the main findings in light of objectives and prior evidence, including fairness where relevant.	Main findings and prior literature are discussed. Formal fairness analysis was not applicable to the synthetic vignette design and was not performed.	pp. 14–18, lines 277–355
Discussion – Limitations	19b	Discuss limitations, bias, uncertainty, and generalizability.	Reported in detail.	p. 18, lines 356–376
Discussion – Context/data challenges	19c	Discuss domain-specific data limitations, representation, missingness, harmonization, and bias where relevant.	Reported for AI-assisted scenario drafting, lack of an independent standard, single-query design, single prompt, model updates, blinding, and pragmatic grey-zone classification.	p. 18, lines 356–376
Discussion – Intended use	19d	Define intended implementation context, end users, autonomy, and human oversight.	Reported as supportive decision support/education under expert supervision, not autonomous use.	p. 5, lines 94–97; p. 19, lines 378–384
Discussion – Input quality	19e	Explain handling of poor/unavailable input data if this is relevant to deployment.	Not applicable: the study did not evaluate real-world deployment or missing/poor-quality clinical inputs.	N/A
Discussion – User interaction	19f	Describe required user interaction and expertise if applicable to the evaluated implementation.	No deployment workflow was evaluated; conclusions explicitly require expert clinical supervision.	p. 19, lines 380–384
Discussion – Future work	19g	State next research steps focused on applicability and generalizability.	Reported, including repeated querying, multicenter expert panels, prespecified safety taxonomy, alternative prompts/model versions, and real-world validation.	p. 18, lines 356–376; p. 19, lines 383–384

Reference: Gallifant J, Afshar M, Ameen S, et al. The TRIPOD-LLM reporting guideline for studies using large language models. Nat Med. 2025;31:60–69. doi:10.1038/s41591-024-03425-5.
