# Supplementary material for: Large Language Models in Perioperative Antithrombotic Management: A Blinded Scenario-Based Expert Evaluation
Source: Diagnostics (Basel). 2026 Sep 4;16(17):2848. doi: 10.3390/diagnostics16172848 (PMC13564537; doi:10.3390/diagnostics16172848)
Supplement: Supplementary file 1 [file diagnostics-16-02848-s001.zip › File S12 Representative Patterns Among Score1 Model Outputs.pdf]

Representative Patterns Among Score-1 Model Outputs

This post hoc analysis is descriptive and non-adjudicated. It summarizes recurring response patterns among outputs that received a score of 1 (completely inappropriate) from one or both evaluators. It was not designed as a formal taxonomy of hallucinations, guideline deviations, critical errors, or potentially harmful recommendations.

A. Recurrent response patterns among score-1 ratings

Model	Domain	Score-1 ratings	Observed recurrent response pattern	Pattern among score-1 ratings
ChatGPT	Anesthesia type	110/194 (56.7%)	Spinal anesthesia recommendation	104/110 (94.5%)
ChatGPT	Clinical rationale	127/200 (63.5%)	Repeated generic statement that coagulation/platelet parameters were appropriate and neuraxial safety was ensured	109/127 (85.8%)
ChatGPT	Proceed/postpone decision	24/200 (12.0%)	Proceed recommendation	22/24 (91.7%)
Claude	Proceed/postpone decision	24/200 (12.0%)	Proceed recommendation	24/24 (100%)
Claude	Anesthesia type	16/200 (8.0%)	Spinal anesthesia recommendation	14/16 (87.5%); 7/8 scenarios
DeepSeek	Proceed/postpone decision	61/200 (30.5%)	Postponement recommendation	58/61 (95.1%)
Qwen	Proceed/postpone decision	56/200 (28.0%)	Postponement recommendation	55/56 (98.2%)

Note: Counts refer to individual evaluator ratings, consistent with the domain distributions reported in Table 2. For the Claude anesthesia-type pattern, the 16 score-1 ratings arose from eight scenarios; seven of these eight scenarios involved a spinal anesthesia recommendation. These cases were outside the 51-scenario common-proceed subset, consistent with the absence of Claude score-1 anesthesia ratings in Table 4. The 'observed recurrent response pattern' column describes the model output associated with score-1 ratings and does not establish causal error type or clinical harm.

B. Representative examples

Model / case	Scenario summary	Model output excerpt	Relevant evaluator ratings
ChatGPT / Case 5	Clopidogrel; TURBT; eGFR 65; platelet count 150; grey-zone scenario.	Interruption: 3 days; Proceed; Spinal. Rationale: "Coagulation parameters and platelet count are appropriate; neuraxial safety is ensured."	Timing 1/1; surgical decision 1/1; anesthesia type 1/1; clinical rationale 1/1.
ChatGPT / Case 6	Dabigatran; open colectomy; eGFR 35; platelet count 90; grey-zone scenario.	Interruption: 5 days; Proceed; General. Rationale: "Coagulation/platelet parameters are borderline; general anesthesia was preferred for neuraxial safety."	Surgical decision 1/1; anesthesia type 5/5; clinical rationale 5/5.
Claude / Case 13	Warfarin; total knee arthroplasty; INR 3.2; platelet count 220; grey-zone scenario.	Interruption: 5 days; Proceed; General anesthesia. Rationale: "Current INR is 3.2 and normalization after interruption is uncertain; spinal anesthesia is contraindicated and general anesthesia is preferred."	Surgical decision 1/1; anesthesia type 5/5.
Claude / Case 16	Rivaroxaban; radical prostatectomy; eGFR 55; platelet count 150; grey-zone scenario.	Interruption: 4 days; Proceed; Spinal anesthesia. Rationale: "When the drug is discontinued 4 days before surgery, neuraxial safety is considered adequate; the surgery is suitable for spinal anesthesia."	Anesthesia type 1/1.
DeepSeek / Case 5	Clopidogrel; TURBT; eGFR 65; platelet count 150; grey-zone scenario.	Interruption: 5 days; Postpone. Rationale included: "Postponement is not required for general anesthesia; ... GA is appropriate."	Surgical decision 1/1; clinical rationale 2/2; anesthesia type not applicable because surgery was postponed.
Qwen / Case 30	Rivaroxaban; total hip arthroplasty; eGFR 65; platelet count 260; grey-zone scenario.	Interruption: 3 days; Postpone. Rationale: "DOAC + total hip arthroplasty (high bleeding risk)."	Surgical decision 1/1; clinical rationale 1/1; anesthesia type not applicable because surgery was postponed.

Interpretive caution: These examples were selected after inspection of the score-1 outputs and are intended only to illustrate recurring response patterns in both directions of the surgical decision. They were not independently adjudicated against a prespecified reference standard and should not be interpreted as a validated safety-error taxonomy or as evidence that any individual recommendation was clinically harmful.
